# Handgrip Strength and Cognitive Function among Elderly Koreans: Insights from the Korean Longitudinal Study of Ageing

**DOI:** 10.3390/ijerph19095262

**Published:** 2022-04-26

**Authors:** Keuntae Kim, Hyemi Kim

**Affiliations:** 1 Department of Public Sociology, Korea University, Sejong 30019, Korea; 2 Department of Social Work, Korea University, Sejong 30019, Korea; milormilor1@korea.ac.kr

**Keywords:** handgrip, cognitive impairment, random-intercept logistic regression, KLoSA, Korea

## Abstract

This study aims to assess handgrip strength as a predictor of cognitive decline within men and women in Korea. A random-intercept logistic regression is fit to estimate the status changes in cognitive function throughout all rounds of the KLoSA, a nationally-representative survey of adults aged 45 years and older. Males in the highest quartile were 71.9% less likely to experience cognitive impairment than those in the lowest quartile. The odds of cognitive impairment for men in the third and second quartiles reduced by 62.6% and 60.4% respectively. Similarly, the odds of cognitive impairment for women declined as 72.7%, 63.0%, and 41.8% for fourth, third, and second quartile, respectively, compared with the lowest quartile. These results imply that assessing and monitoring handgrip strength may enable us to identify subgroups of the elderly with higher likelihood of cognitive impairment in Korea.

## 1. Introduction

Maintaining adequate physical functioning and cognitive ability are essential two pillars that buttress a healthy aging process and overall well-being in older adults. Both are crucial determinants for health and mortality, and, in general, they tend to decline in older ages [1,2,3,4]. It is therefore not surprising that a substantial body of research has examined the association between physical functioning and cognitive capability. However, the vast majority of past studies analyzed samples drawn mostly from Caucasian population in Western countries, such as the U.S. [5,6,7], Canada [8,9], U.K. [10,11], and Germany [12]. As a result, it is not well known about whether similar trends are found in non-Western contexts, particularly Korea, where the population is aging and the number of elderly suffering from dementia, including mild cognitive impairment (MCI), has been skyrocketing over the past several decades.

Furthermore, the small number of past studies that directly address the link between physical frailty and cognition in elderly Koreans [13,14,15,16,17,18] relied mostly on cross-sectional analyses. However, this is methodologically problematic because cross-sectional analyses presume that between-person age differences can provide valid proxy estimates for within-person aging-related changes [2]. In other words, cross-sectional analyses are unable to adequately account for heterogeneity that occur within a person across time. Thus, to understand the etiology more thoroughly, the relationship between handgrip strength and cognitive impairment should be studied by employing longitudinal analysis.

To fill this gap in the literature, this study aims to assess handgrip strength as a predictor for cognitive decline within older men and women in Korea. To this end, the present study drew data from a nationally-representative sample of middle-aged and community-dwelling adults at the baseline interview, which has been followed up for 10 years and measured physical functioning and cognition in all waves. This study’s analysis also accounts for important socioeconomic characteristics, including the frequency of social engagement, which would affect both physical frailty and cognitive functioning, in addition to a variety of health related factors, such as regular exercises, obesity, cerebrovascular disease, ADL, and more.

### 1.1. Prevalence of Cognitive Impairment in Korean Elderly

Prolonged human longevity is a global phenomenon, particularly in wealthy nations, though developing countries are rapidly catching up. It was recently estimated that 50 million people suffer from dementia, of which roughly two-thirds have Alzheimer’s disease [19]. However, there is substantial heterogeneity in the prevalence of dementia across regions in the world. The prevalence of dementia in developed countries was estimated as 13.9% in the US, 8.0% in Canada, 6.6% in Britain, and 8.3% in Italy. In developing countries, the prevalence of dementia was estimated to be from 1.8% to 4.0% [19].

**Figure 1:**
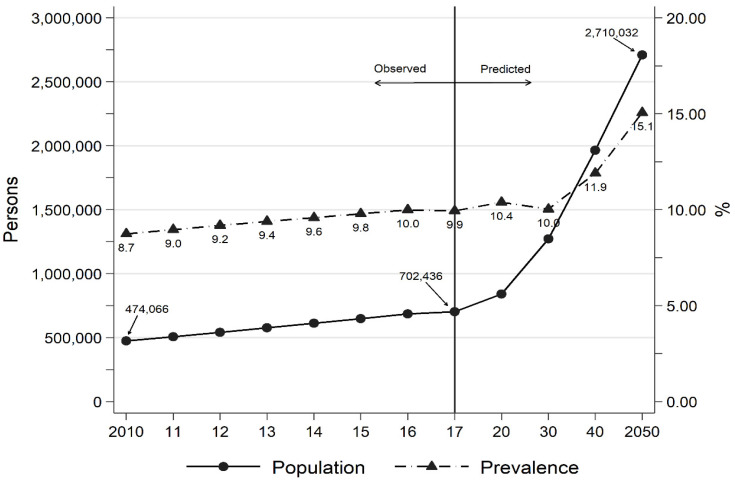
Trends in the Number of Persons with Alzheimer’s Disease and Prevalence Rate in Korea, 2010–2050.

The predicted and observed diagnoses of Alzheimer’s Disease (AD) in Korea, expressed as both a count and as prevalence rate, is illustrated in Figure 1. With its rapidly-aging population, the number of Korean persons with AD is expected to double every two decades: 474,000 patients in 2010 increased to 702,000 in 2017, and is estimated to reach to 2.7 million by 2050 [20]. Furthermore, prevalence of AD was 8.7% in people aged 65 and older in 2010, and is currently about 9.9%. Thus, roughly one in ten persons aged 65 and older in Korea seems to suffer from AD, and this rate is projected to reach at 11.9% in 2040 and 15.1% by 2050.

In addition, the prevalence of AD seems to increase exponentially as an individual ages [21]. According to an epidemiological study of dementia in 2016, the prevalence of dementia was estimated to be 1% among those aged 65–69, 4% in 70–74, 12% in 75–79, 21% in 80–84, and 40% in 85 and older. Hence, the prevalence rate by age roughly doubles with every five years of age [22].

As a result of soaring levels of dementia in aging population, the costs that are associated with the disease—including direct costs for treating patients and indirect costs such as the loss of hours worked by caregivers—have also increased rapidly. More serious is that the economic burden of an increasing number of dementia patients will rise for the foreseeable future.

### 1.2. Determinants of Cognitive Impairment

Cognitive impairment, including AD, is complex and multi-dimensional; age is the strongest risk factor, suggesting that biological senescence is closely associated with the onset and progress of the disease [21], while also implying that AD is a manifestation of other risk factors—including genetic predisposition, health behaviors, and socioeconomic conditions—accumulating over an individual’s life. Except for the effects of age, the nature of the association between muscle strength and cognitive decline is not entirely clear, though an increasing number of studies has addressed the issue [2]. Existing evidence suggests that there are several potential mechanisms for explaining how physical frailty, including handgrip strength, predicts long-term cognitive impairment [21].

First, according to the vascular pathway hypotheses, vascular risk factors, such as obesity, and high cholesterol, smoking, and vascular morbidity (e.g., diabetes, blood pressure, and silent brain infarcts) are significantly associated with the onset of cognitive impairments. Among the causes of cognitive impairment, vascular risk factors are of particular medical and policy concern because they, as opposed to other risk factors, can be controlled to some extent through appropriate intervention or management. In a study that examined the association between vascular risk factors and cognitive impairment among middle-aged Koreans [23], high systolic and diastolic blood pressure, high blood sugar, and low cholesterol level were significantly associated with cognitive impairment even after adjusting for age, past occupations, physical activity ability, and cholesterol level, but this association was not significant in the analysis using stratified educational attainment. Also, there is ample evidence that the experience of cerebral and cardiovascular diseases, such as stroke [24] and silent brain infarcts [25], substantially accelerates the onset of dementia and AD even after taking other risk factors into account. The presence of cerebrovascular disease, even if it does not necessarily cause sizeable deficits itself, might lead to decline in cognitive function by reducing the volume of brain tissue in a concomitant aging process [24]. Relative to other vascular risk factors, however, the effect of being overweight or obese seems to be more complicated. A number of studies, both cross-sectional and longitudinal, found that a high BMI in midlife is closely associated with dementia in later life [26]. Furthermore, evidence from long-term follow-up studies highlighted that rapid increase in BMI in old ages can be a significant predictor for development of dementing disorders [27]. Nevertheless, results for the effect of BMI in later life appear to be mixed. For instance, Fitzpatrick et al. [28] found that BMI and the onset of dementia inversely associated after age 65, whereas Atti et al. [29] reported that low BMI raised the likelihood of dementia among the elderly in similar ages.

Another hypothesis emphasizes the role of psychosocial factors as predictors for dementia and AD. One systematic review of longitudinal studies that examined the effect of social network, physical leisure, and non-physical activity revealed that social, mental, and physical lifestyle components all had positive effects on cognitive function and protective effects against dementia [30]. Also, a recent empirical study in Korea [31] reported that those who participate in religious, leisure, and public activities had a higher—and statistically significant—mean score on cognitive function than non-participants. Further, much research has shown that the adverse effects of an impoverished environment on memory and learning are reversible, and that environmental opportunities for physical activity, learning, and social interaction may prevent or reduce cognitive deficits along the aging process [30]. Related to this is that geographical areas, as a proxy for social, economic, cultural, and environmental characteristics, might also influence dementia among the elderly. Though a number of past studies have compared and contrasted dementia incidence in rural vs. urban areas, the results appear to be mixed in Korea: Kim, Kang, & Yoon [32] compared mean scores of cognitive function, measured with Korea-Mini Mental Status Evaluation (K-MMSE), between the old-age populations residing in rural and urban areas, and found that the performance of rural elderly was lower than that of the urban elderly even after adjusting for sociodemographic and health factors, whereas Shin et al. [33] examined the prevalence of AD among the elderly in rural and urban areas found no significant differences between the groups.

In a third theory, cognitive reserve hypothesis, social and physical activity may enhance an individual’s tolerance to brain diseases through an increase in intellectual activity and more rapid brain recovery and repair [34]. Social engagement could be a mechanism for this theory, in which it suppresses the risk of dementia by reducing stress, which influences a wide range of biologic systems and cardiovascular disease. Moreover, rich social networks and frequent involvement in social activities may also entail greater social capital, which builds material and psychological resources that are beneficial to preventing cognitive decline [35]. It should be noted that there are data on the role of cognitive reserve not only on cognitive function but also on the functional status of older adults [36].

In addition to these potential pathways from physical functioning to cognitive impairment, a large body of past research have found a significant association between socioeconomic status and cognitive function in old-age population. In particular, researchers have repeatedly found that a low level of educational attainment is closely linked with an increasing likelihood of mental disorders in the elderly. For example, Karp et al.’s [37] longitudinal analysis on a sample of Swedes aged 75 and older suggests that a low level of educational attainment significantly elevates the odds of dementia, even when occupation-based SES score was accounted for. Similar findings were reported by Ngandu et al. [38], who controlled for health behaviors in addition to education and SES. Collectively, these results highlight the importance of cognitive reserve, which is positively associated with educational attainment, and is thought to prevent or delay the onset of dementing syndrome [39]. Alternative interpretation would be that educational attainment level may indicate IQ and family environment while growing up, all of which are also related closely with cognitive ability in later life [21].

In sum, as populations have aged globally, the prevalence and incidence of cognitive impairment, including AD, have been increasing significantly—and Korea has followed this pattern. As the result of a skyrocketing number of elderly and a concomitant increase in dementia, a great deal of past studies explored a wide variety of potential risk factors. One of the most prominent, and perhaps most proximate, determinants of dementia and AD in the elderly is vascular diseases and its associated risk factors. Furthermore, evidence suggests that psychosocial factors matter for preventing or delaying dementia and AD. In general, the weight of evidence indicates that physical and cognitive functioning in older adults are closely interwoven [40]. Nevertheless, a considerable number of etiologic pathways from handgrip strength, which Clouston et al. [2] suggested as one of most effective measures for physical frailty, toward dementia in Korea remain unclear, and an empirical analysis using longitudinal methods is therefore warranted.

## 2. Materials and Methods

### 2.1. Data

This study draws data from the Korean Longitudinal Study of Ageing (KLoSA), an ongoing survey of a nationally-representative sample of adults aged 45 years or older since 2006. The primary purpose of the KLoSA is to produce data that measures the social, economic, psychological, demographic, and health status of the elderly, and that establishes effective social and economic policies [41].

In the 2006 baseline interviews, the KLoSA surveyed 10,254 participants, identified by a multistage stratified-area probability sample of households representing the entire population of Korea, except Jeju island [42]. Though the panel attrition rate was not particularly high, the KLoSA augmented the sample by adding a new sample of 920 persons who were born in 1962 and 1963 (aged 51 and 52) in 2014 [41].

The present study uses data from the 1st through 6th waves of the KLoSA (2006, 2008, 2010, 2012, 2014, and 2016), which were available for public use at the time of this writing. All respondents who had data for at least two waves were retained in the analyses. After listwise deletion of missing data, the resulting analytic sample included 4219 males and 5402 females.

### 2.2. Measures

In the present study, the dependent variable is an elderly person’s cognitive impairment. In all rounds of the KLoSA, it first measured respondents’ cognitive functioning using the Korea-Mini Mental Status Evaluation (K-MMSE), which is widely used as a brief screening test for dementia in which scores range from 0 to 30 points. The K-MMSE is composed of eleven questions in five areas of cognitive function: orientation, registration, attention and calculation, memory, and language [13]. The KLoSA used these scores to classify respondents into three groups: normal (greater than 24), declining cognition (between 18 and 23), and close to Alzheimer’s (lower than 17). These cognitive statuses were collapsed into a dichotomous variable (1 if close to Alzheimer’s and 0 otherwise) to fit a random-intercept logistic regression. Because the K-MMSE scores are available in each wave, this measure is treated as a time-varying variable.

Though a variety of elderly physical functioning measures—such as walking speed, chair stands, flamingo stand times, and lung function, etc.—have been suggested in past literature [2], this study uses handgrip strength because data on other measures was lacking. Thankfully, handgrip strength was measured in the same manner in all waves of the KLoSA: the interviewer did not proceed with respondents who did not want to participate or whose hand was injured or sick, but for all others, the subjects squeezed the dynamometer with as much force as possible, with measurements recorded twice for each hand. The highest value among the four test results was recorded as the final handgrip strength for the individual. Since there is a significant difference in handgrip strength between genders among older adults [13,43], the reported handgrip strength was divided into quartiles separately by gender: for men, 1 if less than 25.0 kg, 2 if between 25.0 kg and 29.0 kg, 3 if between 29.0 kg and 32.5 kg, and 4 if greater than 32.5 kg; for women, 1 if less than 14.5 kg, 2 if between 14.5 kg and 17.5 kg, 3 if between 17.5 kg and 20.0 kg, and 4 if greater than 20.0 kg.

The present study also accounted for health-related risk factors that have been reported to contribute to cognitive impairments among the elderly [44,45,46]; these include obesity, diabetes, cerebrovascular disease, activities of daily living (ADL), and regular exercises. Variables for cigarette smoking and heavy drinking were initially considered as behavioral risk factors. However, both variables had substantial missing values, and there were many inconsistent and illogical answers across survey waves (e.g., subjects who reported as “former smoker” at wave 1, but reported as “never smoker” at wave 2). Thus, these variables could not be included in the present analyses. Nevertheless, preliminary analyses that included the smoking and drinking variables yielded similar results as those presented here. In the KLoSA, a respondent’s body weight (kg) and height (m)were used to calculate body mass index (BMI), which was then grouped into five categories: underweight, normal, overweight, obese, and extremely obese; obese and extremely obese were coded as 1, and all others as 0. In each wave, respondents were asked if a doctor diagnosed any of several chronic diseases in the previous year, including diabetes and cerebrovascular disease; a “yes” response was coded as 1, and a “no” as 0. ADLs are routine activities people do every day without assistance [47], and those were measured in seven ways in the KLoSA: eating, bathing, personal hygiene, getting dressed, toileting, transferring, and maintaining continence. ADL are usually expressed as number of preserved or lost function, and a higher ADL value indicates a greater need for help in performing basic activities. In each wave, regular exercise was coded 1 if the respondent did physical exercises regularly at least once a week in the past year, and 0 otherwise.

The analysis included several additional control variables that have been significantly associated with both handgrip strength and cognition in previous studies [4], consisting mainly of socioeconomic background measures: highest grade completed, total household income, marital status, region of residence, and social engagement. At each wave, respondents were asked to indicate the highest level of educational attainment they achieved, and the answer was measured with four categories (1 = elementary school or lower, 2 = middle school, 3 = high school, and 4 = college or higher). Though, in theory, the highest grade completed can change at any time throughout one’s life course, we treated it as a time-constant variable by using educational attainment reported at the baseline interview because the rate of adults returning to school is substantially lower in Korea than in other developed nations [48].

Total household income was defined as the total income of all household members who live together in the past year. Because all available household members were included in the survey, it is possible that different member of the family may report different estimates for the total household income. When this arises, the reported value from the householder, who presumably knows the household income and assets best, was accepted. This variable was grouped into quartiles within each wave.

Marital status of the respondent was measured in each wave with four categories: never married, married, divorced/separated, and widowed. Preliminary analysis indicated that the vast majority of the KLoSA respondent were currently married at each wave (roughly 92% of males and 66% of females); this variable was coded 1 if married and 0 otherwise. The region of residence was measured with three categories: metropolitan areas, small- or medium-sized cities, and rural areas. Social engagement was measured with ten categories for the frequency of meeting with close friends (1 = almost everyday, 2 = once a week, 3 = two or three times a week, 4 = once a month, 5 = two or three times a month, 6 = once a year, 7 = three or four times a year, 8 = five or six times a year, 9 = a few times a year, and 10 = no close friends to meet). This variable is reverse-coded so a higher value reflects more frequent interactions with friends.

### 2.3. Analyses

This study employed a random-intercept logistic regression to estimate status changes in cognitive functioning throughout the KLoSA waves. This analytic strategy was chosen because an ordinary logit model makes the unrealistic assumption that the responses for a given respondent are conditionally independent given the covariates [49]. The random intercept can be regarded as the unobserved heterogeneity, or the combined effect of omitted subject-specific (i.e., time-constant) covariates that cause some subjects to be more prone to cognitive impairment than others.

Rather than including interaction terms between independent variables and gender, the analysis fit separate models by gender examining associations between handgrip strength and cognitive impairment. This was done because physical capability, including handgrip strength, may differ substantially among the elderly even after adjusting for age and body size [50]. It should be noted that we cannot directly compare the absolute magnitudes of coefficients across gender, as each model has different constants; we can only interpret the patterns of relative differences.

## 3. Results

Descriptive statistics for all variables in the analysis, delineated by gender, are presented in Table 1. Consistent with global gender trends for dementia, including AD [19], a substantially higher fraction of females in the KLoSA appear to suffer from cognitive impairments than their male counterparts. For example, roughly 11% of women are suspected to have impaired cognition, compared to about 4% of males. However, the distributions of handgrip strength by gender were similar: roughly half of the subjects were in the highest quartile and about one seventh were in the lowest quartile.

The mean age of the respondents was roughly 60.6 and 60.7 for males and females, respectively, and handgrip strength for about 70% of males and females fall onto the third and fourth quartiles. Roughly one-quarter of men and women in the sample were either obese or extremely obese, while approximately one in ten subjects had diabetes. Older Koreans seem to meet close friends 4–5 times per year on average and, consistent with gender differences in social skills, the mean score for social engagement was slightly higher for females than for males.

Results from the random-intercept logistic regression predicting cognitive impairment are presented in Table 2. Overall, in line with findings from past studies [13], handgrip strength was significantly associated with the odds of cognitive impairment in both sexes, even after accounting for other relevant risk factors. Furthermore, quartiles of handgrip strength and the odds of cognitive impairment indicated a negative gradient relationship: those with higher handgrip strength show a lower odds ratio of cognitive impairment compared to those with lower levels of handgrip strength. More specifically, men in the second quartile of handgrip strength are 60.4% less likely to experience cognitive impairment than those in the first quartile; these differences are 62.6% and 71.9% for men in the third and fourth quartiles, respectively. For females, it appears that cognitive decline gradient is steeper than that for males: the odds for being close to Alzheimer’s decreases by 41.8%, 63.0%, and 72.7% in each successive quartile.

With respect to health risk factors, as found in prior research [51], cerebrovascular disease had a significant impact on the odds of cognitive impairment, whereas obesity and diabetes showed negligible effects. The results also suggest that the effect of cerebrovascular disease on cognitive impairment is much more salient among males than among females: it elevated the odds of cognitive impairment by approximately 3.4 times among males but only 1.7 times among females.

In general, as suggested in prior research [52], higher educational attainment appears to lower the odds of cognitive impairment compared with those having completed elementary education. This may reflect that higher socioeconomic status is closely associated with greater cognitive reserve and physical functioning in later life [53]. However, somewhat unexpectedly, the protective effect of education among those with college education is least pronounced—and not statistically substantial—when compared to those with middle or high school education; this is true for both men and women. A similar pattern was observed among the quartiles of household income: among both men and women, while the second and third quartile showed significantly lower odds ratio of cognitive impairment than those in the first quartile, but the coefficient for the highest quartile lost statistical significance.

With respect to the area of residence, the results indicate that living in rural areas or small- and medium-sized cities significantly raises the odds of cognitive impairment in men and women, net of pertinent confounders. Older males in small or medium cities are about 45.8% more likely to experience cognitive impairment than those in metropolitan areas, and older females experienced increases of 36.4% and 46.5% in medium cities and rural areas, respectively. This result is in accordance with Kim et al. [32], who found significantly lower cognitive function scores among the elderly in rural areas compared with their counterparts in metropolitan areas in Korea.

In terms of social engagement, greater frequency of meeting with close friends significantly diminishes the likelihood of cognitive impairment among both men and women. More specifically, a one-unit increase in social engagement decreased the odds of cognitive impairment by 8.7% for males and 5.5% for females. However, the protective effect of being currently married was found only among females, perhaps because females are significantly less likely to be currently married than male counterparts due to their longer life expectancy.

As found in past literature [54], the results suggest that regular physical exercises significantly reduce the risk of cognitive impairment. Net of other factors, the odds of cognitive impairment for regularly-exercising males diminished by 40.6% compared to those who do not exercise regularly; for females, on average, regular exercise reduced the odds of cognitive impairment by 41.6%. Furthermore, given the close association between physical functioning and cognitive impairment [1], it is not surprising that ADL significantly elevates the odds of cognitive impairment. The results suggest that one more ADL limitation increases the odds of cognitive impairment by 36.1% and 36.9% for males and females, respectively.

**Figure 2:**
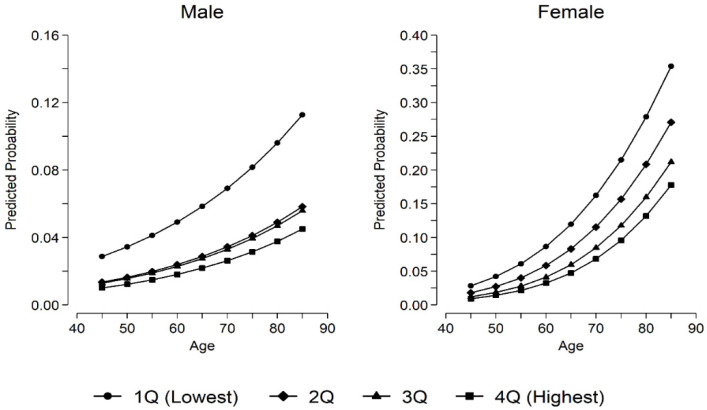
Predicted Probability of Being Close to Alzheimer’s by Gender and Handgrip Strength Quartiles: Korean Longitudinal Study of Aging (KLoSA).

Figure 2 illustrates the predicted probabilities of experiencing cognitive impairment (i.e., close to Alzheimer’s) by quartiles of handgrip strength and age, delineated by gender. This graph is derived from the estimated coefficients in Table 2, with all covariates except handgrip strength and age calibrated to their mean. Consistent with past research [21], the overall shape of the curves suggest that the probabilities increase exponentially with age for among men and women.

Furthermore, in both men and women, the rate of increase in the probability of cognitive impairment was greatest among the lowest quartile of handgrip strength while the same probability for other quartiles increased comparatively more slowly. As a result, discrepancies in the probability of cognitive impairment by the quartile of handgrip strength grew over time. However, the growing discrepancy was much more pronounced in women than in men. In case of males, only those in the lowest quartile showed a significantly higher probability of cognitive impairment while discrepancies among other quartiles were trivial. By contrast, in women, clear gradients by handgrip strength were observed in all ages, and the increase in the probability of cognitive impairment was largest in the lowest quartile (it increased 12.5 times from age 45 and age 85) compared with the highest quartile (it increased 19.4 times between age 45 and 85).

## 4. Discussion

It appears that research on the association between handgrip strength and health outcomes in the elderly Korean population is still in its infancy. To fill in the gap in the literature, this study examined longitudinal associations between handgrip strength and cognitive impairment, after adjusting for relevant confounders by using a large and nationally-representative sample of older Koreans from the KLoSA data. Furthermore, given important gender differences in psychosomatic health outcomes, this article explored whether and how handgrip strength can vary by gender. Hence, this study contributes to the extant literature by allowing the effects of handgrip strength to vary across gender. Also, by dynamically examining the association between physical frailty and cognitive impairment over time, we can gain insight on the changing gradient for cognitive capacity and enhance our understanding on the pathophysiological aspects of cognitive impairment among older adults in Korea. 

Analyses using random-intercept logistic regressions indicate that quartiles of handgrip strength are not only significantly associated with the odds of cognitive impairment, but also correlated in a gradient fashion. More specifically, males in the highest strength quartile were 71.9% less likely to experience cognitive impairment than those in the lowest quartile; the odds of cognitive impairment for men in the second and third quartiles were lower by 60.4% and 62.6%, respectively. Similarly, the odds of cognitive impairment for women declined by 41.8%, 63.0%, and 72.7% in the second, third, and fourth quartiles, respectively. Hence, these results imply that physical functioning may have significant and independent effects on cognitive capability in elderly Koreans, even after accounting for pertinent risk factors.

Among health-related risk factors, experiencing a cerebrovascular disease appears to substantially increase the onset of cognitive impairment, though the effect was more pronounced among males than females. More specifically, a diagnosis of cerebrovascular disease increased the odds of cognitive impairment about 3.4 times for men and 1.7 times for women, net of other factors. As suggested by a large body of neuropathological studies, cerebral and cardiovascular diseases may lead to additional damage to aging brains and facilitate clinical dementia [34]. However, in contrary to expectation, the effects of diabetes and obesity in this study were either trivial or inconsistent between genders. These results might be attributable to the relatively short follow-up periods in which obesity or diabetes have not yet had enough time before the onset of cognitive impairment. Alternatively, the effects of BMI or diabetes were mediated by other covariates in the model, such as ADL or regular exercises.

Despite its important findings, there are several limitations to the present study. First, a number of instruments that can measure physical functioning among the elderly, such as walking speed, chair stands, lung function, flamingo stands, have been developed and examined extensively [54] but were not examined here. Also, various alternative domains of cognition, such as fluid cognition, crystallized cognition, and mental state, can be utilized for analysis. Furthermore, it is possible that not all measures of physical and cognitive functioning were equally associated [2,55]. For instance, one meta-analysis found that handgrip strength was associated with mental state while walking speed was correlated with fluid cognition [2]. Unfortunately, because of the absence of these measures in the KLoSA, only a single dimension of physical and cognitive functionality could be examined. However, given the robust effect of handgrip strength on cognitive impairment, it is highly unlikely that the main findings here would differ substantially.

Second, though specific causal mechanisms of intergenerational transmission of mental illness, including AD, have yet to be studied more thoroughly, there is ample evidence that AD and other mental disorders may be heritable to some extent. For instance, Green et al. [56] examined more than 20,000 family members and relatives of AD patients in 17 institutions, and found that first-degree relatives of AD patients are substantially more likely than non-AD relatives. They also reported that genetic heritability might be higher among African-Americans than among Whites. Based on these results, Qiu et al. [21] proposed genetic predisposition as an important predictor for onset of AD, but the present study was not able to incorporate measures that represent genetic predispositions because the KLoSA lacked this information. However, these factors should be pursued as an important avenue for future research.

Finally, numerous epidemiologic studies have found significant effects of hormones or nutrition on both physical frailty and cognitive functioning. In particular, Maggio et al. [57] found that testosterone may have a protective effect on cognitive decline among the elderly by inhibiting accumulation of amyloid beta protein. In addition, a decrease in testosterone seems to facilitate depletion of muscle mass [58], which, in turn, leads to cognitive impairment. Despite the important roles that hormones might play in affecting frailty and cognitive function, the present study was not able to examine those factors owing to data limitation. Given the evidence for an association between these factors and cognitive impairment, it is an area which merits further investigation.

## 5. Conclusions

In conclusion, this study employed handgrip strength as a proxy for physical frailty, and explored its effect of on cognitive decline in elderly Koreans. The results implied that lower handgrip strength significantly enhances the odds of cognitive impairment in both men and women, even after other potential risk factors are taken into account. These results offer promise because simply assessing and monitoring handgrip strength may enable us to identify subgroups of the elderly with a higher likelihood of experiencing mental disorders, including AD. At the same time, more rigorous data collection and analyses that can illuminate etiologic pathways between handgrip strength and cognitive impairment should be carried out. Also, despite still few, the mutual interaction between cognitive status and physical/motor performance is already a topic of interest [59], and future research should pursue the possibility of reciprocal relationship between physical frailty and cognitive impairment more extensively.

## Figures and Tables

**Table 1:** Sample Means and Standard Deviations by Gender for Variables Used in an Analysis of Cognitive Impairment: Korean Longitudinal Study of Aging (KLoSA) 2006.

	Male		Female	
	*n*	%	*n*	%
*Cognitive Ability*				
Normal	3708	96.4	4224	88.8
Close to Alzheimer	140	3.6	533	11.2
*Handgrip Strength*				
1Q	549	14.3	713	15.0
2Q	571	14.8	696	14.6
3Q	783	20.4	1030	21.7
4Q	1945	50.6	2318	48.7
*Obesity*				
No	3011	78.3	3630	76.3
Yes	837	21.8	1127	23.7
*Diabetes*				
No	3380	87.8	4230	88.9
Yes	468	12.2	527	11.1
*Cerebrovascular Disease*				
No	3734	97.0	4655	97.9
Yes	114	3.0	102	2.1
*Highest Grade Completed*				
Elementary School	1177	30.6	2653	55.8
Middle School	669	17.4	795	16.7
High School	1336	34.7	1078	22.7
College+	666	17.3	231	4.9
*Household Income*				
1Q	839	21.8	1244	26.2
2Q	892	23.2	1164	24.5
3Q	979	25.4	1146	24.1
4Q	1138	29.6	1203	25.3
*Married*				
No	260	6.8	1367	28.7
Yes	3588	93.2	3390	71.3
*Region of Residence*				
Metropolitan Areas	1642	42.7	2105	44.3
Small or Medium City	1306	33.9	1553	32.7
Rural Areas	900	23.4	1099	23.1
*Regular Exercises*				
No	2182	56.7	2986	62.8
Yes	1666	43.3	1771	37.2
	Mean	SD	Mean	SD
*Age*	60.61	10.24	60.67	10.89
*Social Engagement*	7.46	2.72	7.71	2.77
*Activities of Daily Living (ADL)*	0.07	0.58	0.06	0.45

*Note*: Sample means are unweighted. Due to rounding, some proportions do not add up to one.

**Table 2:** Parameters and Standard Errors of the Random-Intercept Logistic Regression of Cognitive Impairment: Korean Longitudinal Study of Aging (KLoSA).

	Male		Female	
	OR	SE	OR	SE
*Handgrip Strength*				
2Q	0.396 ***	(0.053)	0.582 ***	(0.049)
3Q	0.374 ***	(0.053)	0.370 ***	(0.036)
4Q	0.281 ***	(0.040)	0.273 ***	(0.026)
*Age*	1.047 ***	(0.007)	1.102 ***	(0.006)
*Cerebrovascular Disease*	3.428 ***	(0.608)	1.693 ***	(0.300)
*Obesity*	1.058	(0.146)	0.939	(0.082)
*Diabetes*	0.767 *	(0.111)	1.122	(0.113)
*Education*				
Middle School	0.406 ***	(0.070)	0.288 ***	(0.045)
High School	0.461 ***	(0.069)	0.386 ***	(0.059)
College+	0.484 ***	(0.094)	0.494 **	(0.144)
*Household Income*				
2Q	0.806 *	(0.099)	0.740 ***	(0.061)
3Q	0.671 ***	(0.096)	0.739 ***	(0.072)
4Q	0.843	(0.130)	0.856	(0.093)
*Married*	0.828	(0.145)	0.643 ***	(0.057)
*Social Engagement*	0.913 ***	(0.015)	0.945 ***	(0.012)
*Region of Residence*				
Small or Medium City	1.458 ***	(0.187)	1.364 ***	(0.138)
Rural Area	0.901	(0.129)	1.465 ***	(0.154)
*Activities of Daily Living (ADL)*	1.361 ***	(0.074)	1.369 ***	(0.072)
*Regular Exercises*	0.594 ***	(0.064)	0.584 ***	(0.048)
Random Part				
*φ*	2.427		2.993	
*ρ*	0.425		0.476	
Observations	17,817		22,527	
Number of persons	4219		5402	
Log likelihood	−2636.72		−5503.27	

*Note*: Standard errors of odds ratios are in parentheses. The omitted category for handgrip strength is 1Q; the reference category for education is elementary school; the reference category for household income is 1Q; and the omitted category for region of residence is metropolitan areas. *** *p* < 0.01, ** *p* < 0.05, * *p* < 0.1.

## Data Availability

Not applicable.
